# Vertical Transmission of Zika Virus: The Immune–Pathophysiological Interface of the Placenta

**DOI:** 10.1155/jimr/6861528

**Published:** 2026-08-29

**Authors:** Carla de Fátima Silva Menezes, Lívia Carício Martins, Gustavo Batista Ferro, Leticia Vieira Teixeira, Juarez Antônio Simões Quaresma, Pedro Fernando da Costa Vasconcelos, Jorge Rodrigues de Sousa

**Affiliations:** ^1^ Graduate Program in Amazonian Parasitic Biology, State University of Pará, Belém, Brazil, uepa.br; ^2^ Arbovirology and Hemorrhagic Fevers Section, Evandro Chagas Institute, Ananindeua, Brazil, iec.pa.gov.br; ^3^ School of Medicine, State University of Pará, Belém, Brazil, uepa.br; ^4^ Department of Pathology, State University of Pará, Belém, Brazil, uepa.br; ^5^ Department of Pathology, Federal University of São Paulo, São Paulo, Brazil, unifesp.br

**Keywords:** placental circulation, vertical transmission, Zika virus infection

## Abstract

Zika virus (ZIKV) infection represents a critical threat to maternal–fetal health due to its ability to cross the placental barrier, converting this normally protective organ into a direct target of viral infection. This study examines the mechanisms by which ZIKV infects placental cells, including trophoblasts and Hofbauer macrophages, through the engagement of specific cellular receptors such as AXL and T‐cell immunoglobulin and mucin (TIM), while simultaneously evading host immune defenses. Although the placental immune system employs interferon signaling and innate immune cells to restrict viral replication, ZIKV disrupts these pathways, promoting chronic inflammation and structural damage within placental tissue. These morphofunctional alterations impair fetal nutrient exchange and facilitate vertical transmission, leading to severe adverse outcomes such as congenital Zika syndrome (CZS), intrauterine growth restriction, and preterm birth. Importantly, pregnancy outcomes appear to depend on a delicate balance between the timing of maternal infection and the effectiveness of the local immune response. Finally, this study highlights the need for advanced molecular approaches and “omics” technologies to address existing knowledge gaps regarding viral variability and to inform the development of future therapeutic strategies aimed at preventing fetal transmission.

## 1. Introduction

Zika virus (ZIKV), an arbovirus belonging to the genus *Flavivirus* and the family Orthoflaviviridae, was first isolated in 1947 in the Zika Forest of Uganda and remained for decades restricted to sporadic cases in Africa and Asia, where it was predominantly associated with mild clinical manifestations. However, between 2015 and 2016, Brazil experienced an unprecedented epidemic characterized by a high incidence of ZIKV infection and the emergence of severe complications, particularly among pregnant women, culminating in a marked increase in cases of microcephaly and other congenital anomalies, which defined congenital Zika syndrome (CZS) [1–5].

This event marked the transition of ZIKV from a virus historically linked to self‐limited clinical presentations to a pathogen of major global impact, revealing an adaptive and dissemination capacity that had not been previously recognized. The magnitude and repercussions of the Brazilian epidemic were decisive for the World Health Organization to declare ZIKV infection a Public Health Emergency of International Concern in February 2016, highlighting its potential to substantially affect global health, particularly in the context of vertical transmission and maternal–child health. This scenario reinforced the urgency of investigating, from an integrated perspective, the biological, immunological, and structural determinants underlying the virus’s high transmissibility and teratogenic potential [6–9].

ZIKV infection during pregnancy revealed particularly severe consequences, establishing the virus as a pathogen with pronounced neuroteratogenic tropism. Vertical transmission, confirmed by multiple studies, may occur during any gestational trimester, although the risk is markedly higher when maternal infection takes place during the first trimester [10, 11]. This period represents a critical window of fetal susceptibility due to the intense proliferative activity of neural progenitor cells and the immaturity of placental antiviral defense mechanisms. The clinical spectrum of CZS includes microcephaly, intracranial calcifications, ventriculomegaly, ocular abnormalities, motor dysfunction, and delayed neuropsychomotor development [11–13].

Beyond neurological manifestations, studies have demonstrated impaired intrauterine growth, fetal growth restriction, preterm birth, and even fetal demise, suggesting that ZIKV infection affects multiple systems beyond the neuroaxis and reflects widespread disruption of embryonic development. Consequently, understanding how ZIKV interacts with the placenta to produce these outcomes has become central, positioning CZS among the most relevant viral congenital conditions in contemporary public health [14].

The placenta plays a pivotal role as an immunological and physiological interface between the mother and the fetus, functioning as a selective barrier that regulates the transfer of nutrients, gases, and molecules while exerting essential immunoregulatory functions to maintain maternal–fetal tolerance [15, 16]. Evidence indicates that, during infection, the placenta acts not merely as a physical barrier but as an immunologically active tissue capable of modulating antiviral, inflammatory, and metabolic pathways in a manner highly specific to ZIKV. A comprehensive understanding of the immunological mechanisms and physiopathological changes occurring in the placenta during ZIKV infection is therefore crucial to elucidate the basis of vertical transmission and fetal injury, underscoring the relevance of this study in guiding future preventive and therapeutic strategies [17].

In the context of ZIKV infection, the study of the placenta and its immune response is fundamental as this structure constitutes the primary site of interaction between the virus and the immune system, directly influencing the likelihood of vertical transmission and adverse fetal outcomes [18]. A narrative review is particularly suitable for integrating heterogeneous evidence derived from clinical, experimental, and molecular studies, allowing for the identification of convergent patterns in placental pathogenesis. Thus, understanding the interplay between the virus, the placenta, and the maternal–fetal immune system is essential for bridging findings from molecular biology to observed clinical outcomes [17].

In light of the above, this narrative review aims to discuss the immunological and physiopathological mechanisms involved in the vertical transmission of ZIKV, with an emphasis on the placental interface, seeking to integrate current findings in the literature to deepen the understanding of the processes that facilitate viral passage from the mother to fetus and their clinical consequences. Specifically, this work organizes recent literature into key thematic axes: (1) viral biology and tropism; (2) immune recognition and activation of interferon pathways; (3) placental cellular responses; (4) mechanisms of immune evasion and viral persistence; (5) clinical repercussions and knowledge gaps. In addition, this review seeks to highlight existing gaps in knowledge, identify potential targets for clinical intervention, and provide a foundation to pave the development of preventive and therapeutic strategies aimed at mitigating the impact of ZIKV infection during pregnancy.

## 2. Biology and Tropism of ZIKV

ZIKV belongs to the genus *Flavivirus* within the family Orthoflaviviridae, which also includes other medically important arboviruses, such as dengue virus, yellow fever virus, and West Nile virus. The ZIKV genome consists of a positive‐sense single‐stranded ribonucleic acid (RNA) of approximately 10.7 kb, encoding a single polyprotein that is subsequently cleaved into three structural proteins (capsid [C], precursor membrane [prM/M], and envelope [E]) and seven nonstructural (NS) proteins: NS1, NS2A, NS2B, NS3, NS4A, NS4B, and NS5. Among these, the envelope (E) protein plays a central role in host cell recognition, membrane fusion, and viral entry and constitutes the primary determinant of viral tropism and a major target of neutralization [19–25].

The genomic organization and structural plasticity of ZIKV partially explain its remarkable capacity to adapt to diverse tissue microenvironments. Adaptive mutations in genes associated with viral replication and host–virus interactions have been linked to enhanced infection efficiency in human cells, including placental cells. Accumulating evidence indicates that Asian‐lineage strains acquired mutations that increased their affinity for neural progenitor cells and placental cells, contributing to the emergence of congenital outcomes observed in the Americas. Such adaptations influence particle stability, replication kinetics, and receptor interactions, accounting for pathogenic differences between African and Asian ZIKV lineages [26–34].

Although African lineage strains frequently demonstrate higher cytopathic effects in experimental systems, the epidemiological association between Asian‐lineage strains and CZS suggests that lineage‐specific pathogenicity cannot be explained solely by replication efficiency. Instead, differences in tissue tropism, receptor engagement, and host–virus interaction dynamics likely contribute to the distinct clinical patterns observed across outbreaks. This apparent paradox highlights the importance of interpreting lineage‐specific findings within their biological and epidemiological contexts.

ZIKV tropism encompasses a wide range of cell types, including neuronal cells, endothelial cells, epithelial cells, fibroblasts, dermal lineage cells, immune system cells, and, of particular relevance, human placental cells. Both in vitro and in vivo studies have demonstrated a marked affinity of ZIKV for neural progenitor cells, which partially explains its neuroteratogenic potential and the hallmark features of CZS [28, 35, 36]. Within the placenta, ZIKV exhibits tropism for trophoblasts, endothelial cells of placental villi, and Hofbauer cells (fetal macrophages), highlighting multiple routes of viral dissemination within the maternal–fetal compartment. Infection of these cell populations supports local viral replication and may compromise placental structural integrity, thereby facilitating viral access to the fetal [36, 37].

**Figure 1:**
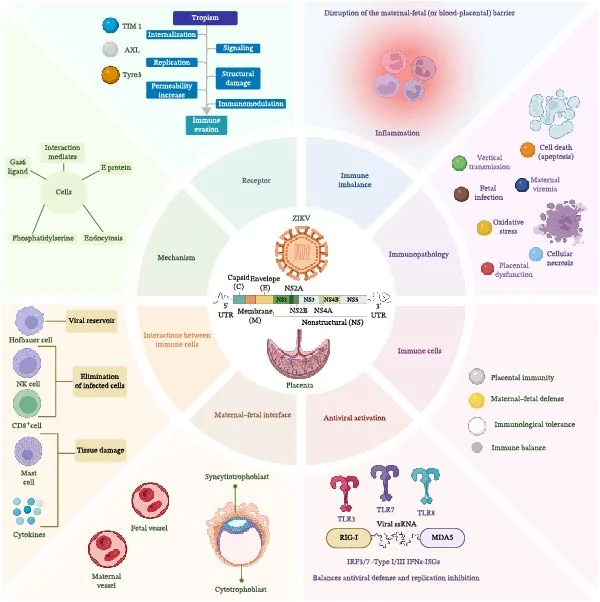
Mechanisms of placental infection by Zika virus and immune responses at the maternal–fetal interface. The central panel illustrates the structural components of ZIKV and the placenta. The virus exploits cellular receptors, including TIM‐1, AXL, and Tyro3, as well as phosphatidylserine‐, Gas6‐, and viral E protein‐mediated mechanisms to promote viral internalization, replication, and immune evasion. Infection triggers antiviral activation through viral RNA sensors, including TLR3, TLR7, TLR8, RIG‐I, and MDA5, leading to Type I/III interferon and interferon‐stimulated gene (ISG) responses. At the maternal–fetal interface, ZIKV disrupts placental structures such as syncytiotrophoblasts, cytotrophoblasts, and maternal/fetal vessels, promoting inflammation, immune imbalance, and placental barrier disruption. Interactions among placental immune cells, including Hofbauer cells, NK cells, CD8^+^ T cells, and mast cells, contribute both to antiviral control and tissue damage. These events ultimately favor vertical transmission, fetal infection, oxidative stress, apoptosis, cellular necrosis, and placental dysfunction (created in https://BioRender.com).

Viral entry into host cells involves interactions between the E protein and host surface molecules such as AXL receptor tyrosine kinase (AXL), TYRO3 protein tyrosine kinase (TYRO3), T‐cell immunoglobulin and mucin‐domain‐containing‐1 (TIM‐1), dendritic cell‐specific intercellular adhesion molecule‐3‐grabbing non‐integrin (DC‐SIGN), and heparan sulfates. Although AXL was initially proposed as a key receptor mediating placental infection, subsequent studies demonstrated that ZIKV can infect placental and neural cells even in the absence of AXL, indicating the presence of redundant or cooperative entry pathways. Accordingly, genetic deletion or pharmacological blockade of AXL reduces, but does not abolish, viral infection, supporting the cooperative involvement of additional receptors such as TYRO3, TIM‐1, and DC‐SIGN, as well as interactions mediated by heparan sulfates (Figure 1) [38–41]. Collectively, ZIKV placental tropism arises from the integration of the receptor repertoire, viral structural plasticity, gestational stage–dependent cellular susceptibility, and tissue‐specific microenvironmental factors, highlighting that placental permissiveness results from multiple converging structural and cellular determinants rather than a single molecular pathway, thereby enabling the virus to cross physiological barriers and establish infection within the fetal compartment.

## 3. Placental Immune Recognition

The maternal–fetal interface constitutes a highly specialized immunological microenvironment that must simultaneously sustain fetal tolerance and maintain surveillance against pathogens. Beyond serving as a physical barrier, the placenta has functions as an immunologically active organ capable of detecting viral invasion through innate immune sensing mechanisms [42]. Recognition of ZIKV infection is primarily mediated by pattern recognition receptors (PRRs) expressed in trophoblasts, decidual cells, and placental macrophages (Hofbauer cells). Viral single‐stranded RNA is detected by endosomal Toll‐like receptors (TLRs), particularly TLR 3 (TLR3) and TLR 7 (TLR7), as well as cytoplasmic RNA sensors such as retinoic acid–inducible gene I (RIG‐I) and melanoma differentiation‐associated protein 5 (MDA5). Activation of these receptors initiates downstream signaling cascades involving adaptor molecules such as mitochondrial antiviral‐signaling (MAVS) protein and TIR‐domain‐containing adapter‐inducing interferon‐β (TRIF), leading to transcriptional activation of antiviral and inflammatory genes [43]. Hofbauer cells, the resident fetal macrophages of the placenta, play a central role as sentinel cells during early infection. Upon viral recognition, these cells rapidly activate innate signaling pathways and produce and deliver proinflammatory cytokines and antiviral mediators. Trophoblasts similarly contribute to early immune sensing, although their responsiveness varies according to the gestational stage and cellular differentiation status [42, 44–46]. In addition to PRR–mediated signaling, inflammasome activation has also been described in placental cells during ZIKV infection, particularly involving the NLRP3 inflammasome, which promotes caspase‐1–dependent processing of interleukin‐1 beta (IL‐1β) and interleukin‐18 (IL‐18), contributing to localized inflammatory amplification. While this response may restrict viral replication, excessive activation can disturb placental homeostasis, highlighting the need for a balanced innate immune response at the maternal–fetal interface [42, 44].

Recognition of ZIKV at the maternal–fetal interface is mediated by PRRs expressed in trophoblasts, decidual cells, and Hofbauer cells. Viral single‐stranded RNA is detected by endosomal TLRs (TLR7/8) and by cytoplasmic RIG‐I–like receptors (RLRs; RIG‐I and MDA5). Activation of these sensors initiates signaling cascades involving adaptor molecules such as MAVS and TRIF, leading to the transcriptional activation of antiviral genes and proinflammatory mediators. Experimental evidence indicates that PRR engagement is influenced by viral strain, inoculum dose, and gestational timing. Distinct ZIKV variants differentially modulate RIG‐I and MDA5 activation, generating heterogeneous innate immune signatures across the decidua, placenta, and fetal tissues. In murine models, these differences correlate with the severity of placental inflammation and fetal growth restriction, suggesting that early viral sensing shapes subsequent infection dynamics [47, 48].

In addition to PRRs, receptors from the TYRO3–AXL–MERTK (TAM) and T‐cell immunoglobulin and mucin (TIM) families exhibit dynamic expression patterns during infection. Although their role in viral entry has been previously described, emerging evidence suggests that these receptors may also contribute to the modulation of innate signaling pathways during ZIKV infection. Spatial redistribution of AXL and related receptors within trophoblastic layers suggests context‐dependent regulation of susceptibility and immune activation [18, 49]. Collectively, these findings indicate that placental immune recognition of ZIKV is a highly complex contextual process determined by viral genotype, gestational stage, and cellular differentiation status. The magnitude and localization of early innate sensing responses represent critical determinants of downstream immune activation and infection outcomes [18, 37, 50].

## 4. Cellular Responses and Immune Modulation During ZIKV Infection

### 4.1. Hofbauer Cells: Viral Reservoirs and Inflammatory Amplifiers

Hofbauer cells, the resident fetal macrophages within the chorionic villi, represent central mediators of placental ZIKV pathogenesis. Multiple histopathological analyses of placentas from infected pregnancies demonstrate hyperplasia, increased cytoplasmic volume, and inflammatory activation of these cells, frequently accompanied by detectable viral RNA and structural proteins. Their strategic localization between maternal and fetal circulations positions them as both immune sentinels and potential facilitators of vertical transmission [51–55].

In vitro and ex vivo models consistently show productive ZIKV infection of primary placental macrophages, with sustained viral replication and release of infectious particles. Although these cells mount antiviral responses, viral clearance is often incomplete, supporting the hypothesis that Hofbauer cells may function as persistent viral reservoirs. However, most evidence derives from short‐term culture systems or late‐gestation placental samples, limiting extrapolation to early pregnancy when fetal vulnerability is the greatest. Persistent activation of Hofbauer cells is associated with increased oxidative stress, extracellular matrix remodeling, and stromal inflammation. While these processes may initially restrict viral dissemination, prolonged activation may compromise villous integrity and facilitate viral access to fetal capillaries. Thus, Hofbauer cells exhibit a dual role: protective containment in the early stages versus contribution to structural dysfunction when activation becomes sustained [36, 51, 56, 57]. The comparative roles of placental cell types, dominant immune mechanisms, and associated clinical implications are summarized in Table 1.

**Table 1:** Placental cell susceptibility, mechanisms, and clinical impact in ZIKV infection.

Placental cell type	Main mechanism of involvement	Interferon response profile	Placental consequence	Associated clinical outcome	References
Cytotrophoblasts (CTB)	Increased permissiveness	Inducible IFN‐I; variable IFN‐III	Barrier disruption; apoptosis	Early vertical transmission	[37, 58–62]
Syncytiotrophoblasts (STB)	Relative resistance; limited entry	Enhanced IFN‐λ–associated signaling	Microvilli alteration; partial containment	Reduced but not absent transmission	[39, 49, 59, 60, 63]
Hofbauer cells	Viral reservoir; inflammatory amplification	IFN‐I production; cytokine release	Stromal inflammation; barrier weakening	Placental insufficiency; fetal exposure	[36, 51, 53, 56, 58, 64]
Decidual macrophages	Immune modulation	Proinflammatory signaling	Local immune imbalance	Variable fetal susceptibility	[10, 44, 65, 66]
Endothelial cells	Vascular activation	IFN–mediated signaling	Increased permeability; hypoperfusion	IUGR; fetal hypoxia	[40, 41, 55, 67, 68]

A summary of placental cell–specific susceptibility patterns, interferon responses, and associated clinical implications during ZIKV infection. Mechanistic interpretations reflect integrated evidence from in vitro, ex vivo, and in vivo experimental models.

### 4.2. Decidual NK Cells: Cytotoxic Control Under Regulatory Constraints

Decidual natural killer (dNK) cells constitute a specialized immune subset responsible for vascular remodeling and local immune regulation during pregnancy. Experimental studies indicate that dNK cells can recognize ZIKV‐infected trophoblasts through stress‐induced ligands and exert selective cytotoxic activity mediated by perforin and granzyme B, thereby limiting local viral spread [57]. Importantly, this cytotoxic activity appears to be tightly regulated. Nonhuman primate models demonstrate that during acute infection, decidual leukocytes display modest inflammatory activation with the preservation of regulatory cytokine profiles [65]. This controlled response suggests that antiviral activity is constrained to prevent excessive tissue damage, reflecting the delicate balance between viral control and maintenance of fetal tolerance [65].

Nevertheless, the magnitude of dNK–mediated protection likely varies with gestational timing and host immune background, factors that remain insufficiently characterized in human studies. The absence of large longitudinal human datasets limits definitive conclusions regarding the protective versus pathogenic contribution of dNK responses [57, 65].

### 4.3. Mast Cells: Early Inflammatory Amplifiers

Placental mast cells contribute to gestational immune regulation and vascular dynamics. During ZIKV infection, primary human mast cells and human mast cell line‐1 (HMC‐1) cell lines demonstrate susceptibility to infection, accompanied by marked degranulation and release of proinflammatory mediators such as tumor necrosis factor alpha (TNF‐α), interleukin‐6 (IL‐6), and interleukin 8 (IL‐8). Ultrastructural analyses reveal mitochondrial alterations and membrane rearrangements consistent with viral replication. These findings suggest that mast cells may function as early inflammatory amplifiers, enhancing local immune activation. However, evidence is primarily derived from in vitro systems, and the in vivo relevance of mast cell–driven pathology during human gestation remains incompletely defined. Excessive mast cell activation may contribute to vascular dysfunction and increased tissue permeability, potentially favoring vertical transmission. Whether this response predominantly restricts infection or promotes placental injury likely depends on infection timing and local regulatory mechanisms [17, 67].

### 4.4. T Cells and Adaptive Immunity

Adaptive immune responses contribute to systemic viral control during pregnancy. ZIKV‐specific CD4^+^ (cluster of differentiation 4‐positive) and CD8^+^ (cluster of differentiation 8‐positive) T cells, together with neutralizing antibodies, have been detected in infected pregnant women, and immunoglobulin G (IgG) antibodies are efficiently transferred to the fetus, providing passive neonatal protection. Cross‐reactive CD8^+^ T‐cell responses induced by prior dengue virus exposure may confer partial protection against ZIKV infection and vertical transmission in experimental models. This observation highlights the epidemiological relevance of maternal immune history in endemic regions. However, the protective role of adaptive immunity at the placental level remains incompletely defined. Most available evidence reflects systemic immune responses rather than localized decidual or villous immunity. Additionally, the impact of preexisting flavivirus immunity on placental inflammation and fetal outcomes remains an area of ongoing investigation [69, 70].

### 4.5. ZIKV‐Induced Immunological Modulation in the Placenta

ZIKV infection induces complex modulation of placental immunity, characterized by divergent patterns of immune activation and suppression. In vitro and in vivo studies demonstrate that the virus causes structural damage and tissue inflammation while concurrently reducing global placental transcriptional activity, impairing pathways associated with antiviral responses and cellular metabolism. This transcriptional suppression correlates with adverse pregnancy outcomes, including intrauterine growth restriction and increased villous necrosis, indicating the functional collapse of the placental immune environment. Additionally, reduced transcription affects essential cell–cell communication modules, impairing signaling among trophoblasts, macrophages, and endothelial cells, thereby amplifying tissue vulnerability and reducing viral containment capacity [71].

Although immune activation occurs, it is markedly dysregulated. ZIKV induces intense local inflammatory responses, with increased leukocyte infiltration and elevated expression of pro‐inflammatory cytokines such as IL‐6, TNF‐α, and IL‐1β, alongside markers of endothelial damage and trophoblast apoptosis [67, 72]. However, this inflammation does not translate into efficient viral clearance, suggesting that ZIKV exploits immune signaling pathways to sustain replication. The virus promotes selective mitochondrial degradation and modulation of mitophagy and mitochondria‐derived vesicles, facilitating persistence in trophoblastic cells. This imbalance excessive inflammation coupled with insufficient antiviral defense demonstrates that ZIKV selectively reorganizes the immune environment to maintain replication without triggering effective clearance mechanisms [73].

Collectively, these findings indicate that ZIKV acts as an opportunistic immunomodulator, activating innate immune components sufficiently to induce inflammation while suppressing effective antiviral mechanisms, thereby weakening the placental defense. The result is a microenvironment characterized by inefficient hyperinflammation and cellular dysfunction, favoring viral replication and fetal dissemination. This hybrid immunological pattern is a hallmark of congenital ZIKV pathogenesis and distinguishes it from other flaviviruses that do not exhibit the same degree of simultaneous inflammatory activation and antiviral suppression [67, 72, 73].

ZIKV infection of trophoblasts induces profound alterations in gene expression and messenger RNA (mRNA) polyadenylation patterns, affecting pathways related to immune responses, energy metabolism, and membrane integrity. Transcriptomic studies reveal global reprogramming in infected trophoblasts, with downregulation of antiviral genes and upregulation of pro‐inflammatory and cellular stress markers [74, 75]. In discordant twin models for CZS, trophoblasts from affected fetuses exhibit imbalanced immune and metabolic responses, indicating that individual variability in transcriptional responses may determine fetal susceptibility. These findings reinforce the concept that not only viral load but also intrinsic placental response characteristics such as IFN signaling efficiency, metabolic robustness, and mitochondrial integrity define the risk of adverse fetal outcomes [75].

Moreover, ZIKV interferes with epigenetic and transcriptional regulation mediated by human endogenous retroviruses (HERVs) and transcription factors associated with interleukin signaling. Infection increases HERV expression and modulates genes related to IL‐6, IL‐10, and interferon pathways, suggesting that the virus reprograms the trophoblastic genome to favor replication while perpetuating chronic inflammation. This HERV activation represents an underexplored but potentially decisive mechanism in amplifying inflammatory responses and compromising genomic stability in placental cells [76].

Persistent inflammation and gene dysregulation contribute to placental structural damage, including villous stromal necrosis, intervillous edema, and disruption of trophoblastic architecture, observed in isolated cases and in infections associated with maternal complications such as Guillain–Barré syndrome (GBS). Thus, ZIKV infection results in multifactorial placental immunometabolic dysfunction, in which inflammatory activation, gene modulation, and mitochondrial interference converge to disrupt tissue homeostasis and increase the vertical transmission risk. These processes define a self‐sustaining pathogenic cycle in which structural damage, antiviral failure, and metabolic stress mutually reinforce placental deterioration [67].

Table 2 provides an integrated mechanistic framework summarizing key cellular targets, molecular pathways, placental consequences, and fetal outcomes, highlighting the causal links that connect immune modulation to vertical transmission during ZIKV infection.

**Table 2:** Immune–pathophysiological interface of the placenta in vertical transmission of Zika virus.

Domain	Key cells/factors	Core mechanism (causal flow)	Placental implications	Fetal impact	Functional direction	References
Target placental cells	CTB, STB, endothelial cells, Hofbauer cells	AXL/Tyro3 expression ↑ → viral entry ↑ → local replication	Villous inflammation; structural disruption	Fetal circulation exposure	Pathogenic	[36, 37, 51, 53, 54, 56, 77]
Viral entry receptors	AXL, Tyro3, TIM‐1, DC‐SIGN, and GAS6	Phosphatidylserine‐mediated binding → apoptotic mimicry → endocytosis	Increased in placental cells	Increased fetal viral burden	Pathogenic	[22, 39, 40, 49, 63]
Innate immune response	IFN I/III, TLR3 IRF3/7, and NF‐κB	PRR activation → IFN production → ISG induction	Partial antiviral containment; chronic villitis if sustained	Growth restriction; inflammatory milieu	Context‐dependent	[47, 58–60, 64]
Viral immune evasion	NS5, STAT2, autophagy pathways	STAT2 degradation → JAK/STAT inhibition → ISG suppression	Reduced antiviral defenses	Viral persistence; enhanced fetal exposure	Pathogenic	[44, 66, 73, 78]
Histopathological alterations	Hofbauer hyperplasia; villous necrosis; calcifications	Macrophage proliferation → epithelial damage → barrier weakening	Impaired maternal fetal exchange	Hypoxia; fetal growth restriction	Pathogenic	[38, 38, 50, 53, 55, 67, 68]
Mechanisms of placental crossing	Infected Hofbauer cells; trophoblasts	“Trojan horse” migration → transcellular transport → microlesions	Barrier disruption	Direct fetal tissue infection	Pathogenic	[36, 56, 63, 79, 80]
Functional impact of infection	Transporters (ABCG2, P‐gp); mitochondria	Nutrient transport ↓ → ROS ↑ → apoptosis ↑	Placental insufficiency	Malformations; neurological injury	Pathogenic	[41, 71, 71, 72, 81]
Highest‐risk period	First trimester	Immature IFN response + high trophoblast proliferation → permissiveness ↑	Reduced barrier efficiency	Severe neurodevelopmental damage	High vulnerability window	[42, 59, 61, 62]

### 4.6. Comparative Aspects Between ZIKV and Other Pathogens in the Placenta

ZIKV circulates in regions endemic for other flaviviruses, particularly DENV and YFV, creating a complex immunological landscape at the maternal–fetal interface. Although all activate classical interferon‐mediated antiviral pathways and ISGs, comparative analyses reveal substantial differences in the magnitude, timing, and cellular distribution of these responses. In first‐trimester trophoblasts and human monocytic cells, ZIKV induces a comparatively attenuated early antiviral response relative to DENV and YFV, allowing for more sustained replication within placental tissues. DENV elicits robust Type I and III interferon activation, whereas YFV induces an intermediate response associated with moderate inflammatory cytokine production. This hierarchy indicates that ZIKV is particularly adept at evading early sensor signaling, conferring a replicative advantage in tissues most vulnerable to vertical transmission, such as early syncytiotrophoblasts [82]

Comparison among ZIKV, DENV, YFV, and the bacteria *Listeria monocytogenes* underscores how different infectious agents shape specific placental immune responses, ranging from moderate antiviral control to excessive inflammation. Whereas ZIKV promotes a subacute inflammatory state permissive to persistence and vertical transmission, bacterial pathogens such as *Listeria monocytogenes* induce destructive immune responses culminating in early fetal loss. These differences reinforce the uniqueness of ZIKV infection, whose pathogenesis depends on both immune evasion and selective manipulation of placental antiviral pathways. This ecological and immunological interplay highlights that the placental outcome is determined not solely by viral factors but also by the broader infectious and immunological context in which gestation occurs [82, 83].

### 4.7. Viral Persistence and Immune Evasion

A defining feature of ZIKV infection during pregnancy is its capacity to persist within the placental tissue even after clinical resolution and clearance of maternal viremia. Replication‐competent viruses have been detected in human placental mesenchymal stromal cells (hPMCs) weeks to months after the acute phase, with stable viral genomes that remain experimentally infectious and reactivatable [84]. These findings indicate that the placenta can function as a long‐term viral reservoir, sustaining infection independently of detectable systemic circulation. Such compartmentalized persistence underscores the unique immunological environment of the placenta, which may permit viral maintenance under conditions that would otherwise favor clearance in peripheral tissues that remain to be clarified [84].

Persistence appears closely linked to both the immunomodulatory character of the maternal–fetal interface and the relatively low turnover of stromal and trophoblastic cell populations. Comparative studies demonstrate that ZIKV induces reduced cytopathic effects and attenuated cell death pathways in trophoblasts compared with microglial or other permissive cell types, allowing prolonged intracellular residence without immediate structural collapse. This property is particularly relevant in clinically mild or asymptomatic maternal infections, where the absence of systemic symptoms does not necessarily reflect eradication of placental infection. Consequently, delayed or ongoing vertical transmission may occur despite apparent maternal recovery [85].

The capacity for sustained placental infection is intrinsically tied to viral immune evasion strategies. ZIKV interferes with key antiviral signaling cascades, notably the interferon‐dependent JAK/STAT pathway, thereby limiting the induction of antiviral gene programs. Viral NS proteins, especially NS5, mediate selective degradation of signal transducers and activators of transcription 2 (STAT2), disrupting signal amplification downstream of interferon receptors and reducing ISG expression. Additionally, ZIKV modulates upstream antiviral sensors, including RIG‐I, MDA5, interferon regulatory factor 3 (IRF3), and TLR3, attenuating early interferon production. These coordinated disruptions weaken antiviral containment while preserving sufficient cellular integrity to maintain viral replication [78].

Mitochondrial reorganization represents another central mechanism of immune evasion. ZIKV infection alters mitochondrial dynamics, reduces functional MAVS signaling platforms, and induces fragmentation that dampens interferon signaling cascades. Concurrent modulation of mitophagy pathways further contributes to the controlled suppression of antiviral responses while sustaining metabolic activity necessary for viral propagation. Rather than provoking overwhelming inflammation, these strategies establish a state of low‐level, persistent replication within a partially suppressed immune environment. Collectively, ZIKV persistence reflects a finely tuned equilibrium between immune evasion and host cell survival. By attenuating interferon amplification, modulating cytosolic sensors, and limiting apoptotic activation, the virus avoids rapid clearance without triggering destructive inflammation. This silent persistence within placental reservoirs provides a mechanistic explanation for continued fetal risk in the absence of maternal viremia and contributes to the unpredictability of congenital outcomes [78, 85].

Emerging evidence suggests that ZIKV may employ additional mechanisms to enhance persistence and evade host immune responses at the maternal–fetal interface. These include “Trojan horse” strategies, in which infected immune cells such as macrophages facilitate viral dissemination across placental barriers. Furthermore, extracellular vesicles may contribute to the transfer of viral components and modulation of recipient cell responses, potentially supporting immune evasion. Tunneling nanotubes have also been proposed as a mechanism for direct cell‐to‐cell viral transfer, allowing ZIKV to bypass extracellular immune surveillance. Although these pathways remain incompletely characterized in placental ZIKV infection, they are supported by broader evidence of host–viral interactions and immune modulation at the maternal–fetal interface. Together, these mechanisms highlight the complexity of ZIKV persistence and immune evasion within this specialized microenvironment [35, 44, 46].

To move beyond descriptive observations, omics‐based approaches should be strategically integrated to elucidate the molecular mechanisms underlying ZIKV infection at the maternal–fetal interface. Transcriptomic datasets can be used to identify differential gene expression patterns in trophoblasts and Hofbauer cells, particularly those associated with antiviral responses and inflammatory signaling pathways [74, 75]. Proteomic analyses may complement these findings by providing additional insights into functional protein networks and host–virus interactions during infection. In addition, metabolomic profiling can provide insights into cellular metabolic and functional reprogramming during infection, which has been increasingly recognized as a key component of host–virus interactions [58, 86].

Importantly, integrative multiomics approaches combining transcriptomic and regulatory layers, including noncoding RNAs, can enhance the identification of key regulatory hubs and signaling pathways that cannot be captured by single‐layer analyses [75, 87]. The integration of publicly available datasets with clinical metadata, such as gestational stage and pregnancy outcomes, may further improve the translational relevance of these findings. Future studies should prioritize standardized sampling protocols, longitudinal designs, and cell type–specific analyses to better resolve the heterogeneity of placental responses to ZIKV infection. Such strategies will be essential to bridge the gap between large‐scale data generation and the identification of clinically relevant biomarkers and therapeutic targets [75, 87].

### 4.8. Apoptosis, Cell Death, and Immunopathology

While ZIKV persistence in the placental tissue depends on controlled immune evasion, progressive infection ultimately activates programmed cell death pathways that contribute to tissue injury. Caspase‐dependent apoptosis represents a central mechanism in ZIKV–induced placental and neural immunopathology. Experimental studies demonstrate the activation of caspases 8 and 3 through modulation of the apoptosis regulator c‐FLIP (FLICE‐like inhibitory protein), a key inhibitor of extrinsic apoptotic signaling. During infection, ZIKV appears to transiently exploit c‐FLIP to maintain host cell viability and sustain replication, followed by caspase activation that facilitates viral particle release and local dissemination. This temporally regulated balance between survival and apoptosis reflects a strategic viral adaptation that maximizes replication while ultimately promoting the spread [81, 88–91].

Beyond direct caspase activation, ZIKV alters the expression of microRNAs (micro RNAs [miRNAs]) involved in apoptosis regulation and antiviral signaling. Infection is associated with downregulation of survival pathways and impaired expression of genes linked to immune cell recruitment, including those governing lymphocyte chemotaxis. Dysregulation of miRNA networks affecting B‐cell lymphoma 2 (BCL‐2) family proteins, c‐FLIP, and cellular stress mediators amplifies apoptotic signaling and weakens local immune coordination. Consequently, the infected placental tissue exhibits not only enhanced cell death but also reduced capacity for immune‐mediated repair, compounding structural vulnerability [87].

Ultrastructural analyses further reveal extensive morphofunctional alterations in the infected placental tissue. ZIKV induces the disruption of trophoblastic microvilli, dilation of the endoplasmic reticulum, mitochondrial swelling, and membrane fragmentation. Mitochondrial injury, characterized by cristae loss and membrane destabilization, intensifies oxidative stress and reinforces proapoptotic cascades. Additionally, reduced expression of transport proteins such as ATP‐binding cassette subfamily G member 2 (ABCG2) and P‐glycoprotein (permeability glycoprotein 1, ABCB1) indicate compromised placental exchange and diminished protective barrier function, directly affecting nutrient transfer and xenobiotic defense [68].

The cumulative effect of sustained apoptosis, mitochondrial dysfunction, and inflammatory activation is progressive architectural collapse of the chorionic villi and impairment of maternal–fetal perfusion. Murine models demonstrate villous disorganization, reduced vascular integrity, and placental hypoperfusion following infection, illustrating the downstream consequences of persistent cell death and immune dysregulation. Importantly, apoptosis in this context is not an isolated cytopathic event but part of a broader pathological continuum linking chronic low‐grade inflammation, structural deterioration, and increased susceptibility of the fetus to ZIKV invasion. Thus, ZIKV–induced immunopathology represents the structural manifestation of earlier immune evasion and persistence mechanisms. When the balance between controlled replication and cellular survival is disrupted, apoptotic and oxidative processes dominate, eroding placental integrity and facilitating vertical transmission [68].

## 5. Interferon and ISGs Dynamics in Placental ZIKV Infection

Following innate immune recognition, placental cells activate interferon signaling pathways involving both Type I interferons (IFN‐α/β) and Type III interferons (IFN‐λ), leading to the induction of a broad repertoire of ISGs with direct antiviral activity. Studies using first‐trimester human placental samples demonstrate that both interferon types are induced during infection; however, their kinetics and spatial distribution differ significantly. In early gestation, IFN‐λ responses may be delayed, potentially creating a transient permissive window for viral replication before the full establishment of antiviral defenses. The induced ISG repertoire includes antiviral effectors with established activity against ZIKV in trophoblasts, such as ISG20, which degrades viral RNA, and membrane‐associated restriction factors, including interferon‐induced transmembrane protein 3 (IFITM3), which limit viral entry and fusion. Additional mechanisms involve the modulation of viral assembly and replication pathways. Intercellular transfer of antiviral mediators through extracellular vesicles and paracrine interferon signaling further amplifies the antiviral state, establishing a coordinated multicellular barrier along placental villi [58, 59, 86].

Despite these protective mechanisms, accumulating evidence supports the existence of a “Type I interferon paradox” in the placenta. While IFN‐I is essential for viral control, excessive or prolonged activation may induce trophoblastic apoptosis, villous disorganization, and impairment of placental barrier integrity. Experimental studies demonstrate that hyperactivation of IFN‐I signaling disrupts trophoblast growth and differentiation and activates proapoptotic pathways as well, involving cysteine‐aspartic protease 3 and caspase‐9 (cysteine‐aspartic protease 9), downregulation of BCL‐2, and modulation of cellular c‐FLIP, collectively contributing to the loss of villous integrity [36, 60, 92]. In contrast, IFN‐λ signaling appears to exert a more localized and less inflammatory antiviral effect. Its activity is gestational stage–dependent and has been associated with reduced placental and fetal viral replication without inducing the same degree of trophoblastic damage observed with exaggerated IFN‐I responses. This functional distinction suggests that the relative balance between Type IFN‐I and Type IFN‐III signaling represents a critical determinant of infection outcomes [86, 92].

Recent findings further indicate that antiviral signaling is compartmentalized between maternal and fetal tissues. Intrinsic fetal MAVS/Type I interferon signaling restricts infection in specific trophoblastic zones and modulates viral dissemination, highlighting the active contribution of the fetal compartment to placental defense. Distinct ISG expression profiles in placental cluster of differentiation 14‐positive cells (CD14^+^ cells) create localized antiviral microdomains within the villous stroma, reinforcing the concept that antiviral protection is spatially regulated. Collectively, these data support a model in which the timing, magnitude, and compartmentalization of interferon responses determine whether the outcome is protective or pathogenic. Proper synchronization between maternal and fetal compartments appears essential to limit viral dissemination while preserving placental structural integrity. This framework also provides a rationale for exploring interferon‐based interventions, particularly IFN‐λ administration during specific gestational windows, as potential therapeutic strategies [93, 94].

## 6. Clinical Implications and Gestational Outcomes

ZIKV infection during pregnancy is associated with severe placental dysfunction and a broad spectrum of adverse gestational outcomes, including fetal demise, spontaneous abortion, intrauterine growth restriction, and CZS. Histopathological and experimental studies demonstrate that infection compromises both structural integrity and metabolic function of the placenta, affecting nutrient and gas exchange, immune barrier properties, and development [31, 69]. Diffuse villous inflammation, fibrin deposition, trophoblast apoptosis, endothelial injury, and placental hypoperfusion collectively impair fetal oxygenation and correlate with growth restriction and intrauterine fetal death. These findings indicate that placental injury is not merely inflammatory but functionally systemic, simultaneously destabilizing immunological, metabolic, and vascular axes essential for fetal development [41, 61, 71].

Sustained viral replication in trophoblastic and decidual cells directly contributes to placental damage and vertical transmission. In human placental models, ZIKV preferentially infects proliferative first‐trimester cells, particularly cytotrophoblasts and Hofbauer cells, which act as viral reservoirs and dissemination portals [61]. Viral NS1 also plays a critical pathogenic role by degrading extracellular matrix glycosaminoglycans and increasing placental vascular permeability, thereby compromising cellular cohesion and maternal–fetal barrier integrity. These structural and biochemical alterations facilitate viral passage into the fetal compartment and exacerbate neurological outcomes. This constellation of mechanisms reinforces that vertical transmission depends not solely on viral presence but on the combined effects of structural damage, endothelial dysfunction, and breakdown of placental selectivity [62, 79, 95].

Animal models provide additional insights into gestational consequences. In nonhuman primates, ZIKV infection results in multifocal placentitis, villous infarction, fibrin deposition, and reduced placental blood flow, frequently accompanied by fetal hypoxia and malformation [41, 95]. Murine models demonstrate that intact placental innate immunity, particularly macrophage and myeloid cell activity, can partially restrict fetal viral load; conversely, immunodeficient conditions markedly exacerbate vertical transmission and fetal demise; these intermodel differences underscore the central role of placental immune competence in modulating disease severity [96].

Viral persistence within the placenta and fetal tissues further contributes to CZS. Even after initial infection, ZIKV maintains genomic diversity during early vertical transmission and subsequently undergoes population bottlenecks within fetal brain tissues, indicating adaptive viral selection within the fetal environment [64, 80, 97, 98]. This persistence favors irreversible neurological damage, including microcephaly, intracranial calcifications, and cortical abnormalities. Although infection can occur at multiple gestational stages, the most severe outcomes concentrate in the first trimester, when organogenesis coincides with heightened trophoblastic permissiveness and immature interferon‐dependent defenses. Simultaneously, rapid fetal neural proliferation renders the central nervous system particularly susceptible to viral cytopathic and inflammatory damage [38, 63, 66, 77, 99].

Collectively, clinical manifestations of congenital ZIKV infection arise from the convergence of placental structural collapse, immune evasion, chronic inflammation, and viral persistence. The resulting spectrum ranges from early pregnancy loss to severe congenital neurological impairment. This variability reflects the dynamic interplay between viral factors, maternal immune history, placental resilience, and gestational timing, highlighting the multifactorial and context‐dependent nature of ZIKV‐associated fetal compromise [41, 61, 96, 100].

## 7. Knowledge Gaps and Future Directions

Despite significant progress in elucidating the molecular and immunological basis of placental ZIKV infection, several critical knowledge gaps persist. Addressing these unresolved questions is essential to refine mechanistic models of vertical transmission and to translate experimental findings into effective preventive and therapeutic strategies [10, 41, 59, 63].

### 7.1. Unresolved Mechanisms of Vertical Transmission

Although structural disruption, immune dysregulation, and viral persistence have all been implicated in transplacental passage, the precise sequence of events leading to fetal infection remains incompletely defined [36, 41, 59]. It is still unclear whether vertical transmission is primarily driven by trophoblastic barrier breakdown, active viral trafficking by infected Hofbauer cells, endothelial compromise, or coordinated failure of multiple placental compartments [36, 37, 40, 41].

Gestational timing adds further complexity. First‐trimester susceptibility suggests that developmental stage–specific factors, including interferon kinetics, receptor distribution, and metabolic vulnerability, critically shape permissiveness [59–61]. Moreover, the extent to which the fetal compartment contributes to antiviral signaling at the maternal–fetal interface remains insufficiently characterized. Defining these stage‐dependent determinants is essential for identifying predictive biomarkers of fetal risk [41, 46].

### 7.2. Interferon Modulation and Therapeutic Perspectives

The paradoxical role of interferon signaling represents one of the most pressing unresolved issues in ZIKV pathogenesis. While Type I interferons are indispensable for viral restriction, excessive or prolonged activation may promote trophoblastic apoptosis, vascular dysfunction, and placental insufficiency. Conversely, Type III interferons appear to provide localized antiviral protection with comparatively limited inflammatory injury [59, 60, 64].

Future investigations should determine whether temporally controlled modulation of interferon pathways, particularly selective enhancement of IFN‐λ or attenuation of maladaptive IFN‐I amplification, can achieve viral control without compromising placental integrity [60, 64]. Identification of ISG expression signatures associated with either protection or pathology may enable risk stratification and inform targeted therapeutic interventions [58, 59].

### 7.3. Cross‐Immunity in Endemic Regions

In flavivirus‐endemic settings, prior exposure to DENV or other related viruses introduces an additional layer of immunological complexity [63, 78]. Cross‐reactive antibodies and T‐cell responses may confer partial protection or, alternatively, modulate viral entry dynamics and inflammatory amplification. The extent to which antibody‐dependent enhancement or altered interferon signaling influences placental susceptibility remains unresolved [44, 78].

Comprehensive longitudinal cohort studies integrating maternal immune history, serological profiling, placental transcriptomics, and fetal outcomes are required to clarify how cross‐immunity shapes the vertical transmission risk [10, 66]. These insights are particularly relevant for vaccine strategies and public health planning in regions with overlapping flavivirus circulation [63, 78].

### 7.4. Experimental Model Limitations and the Need for Standardization

Current mechanistic insights derive from heterogeneous experimental systems that vary accordingly to viral strains, inoculum doses, gestational timing, and host immune status [41, 63, 64]. Such variability contributes to conflicting findings regarding receptor dependency, interferon‐mediated protection versus pathology, and viral persistence dynamics [39, 60, 64].

Future progress will depend on harmonized methodological frameworks incorporating standardized viral isolations, physiologically relevant inoculum conditions, and coordinated reporting of the gestational stage [63]. Integration of human placental organoids, longitudinal clinical cohorts, and finally, but not least, multiomics approaches will be critical to improve reproducibility and enhance translational relevance [10, 59]. A unified experimental strategy will facilitate the identification of predictive biomarkers and the development of targeted maternal–fetal interventions [58, 64].

## 8. Critical Appraisal of Experimental Models and Evidence

While the preceding sections describe multiple molecular and cellular mechanisms underlying ZIKV placental infection, it is essential to critically evaluate the experimental frameworks from which these conclusions derive. Although substantial progress has been achieved, the available evidence is marked by methodological heterogeneity that significantly influences the interpretation of mechanistic findings and translational implications [41, 59, 63, 64].

### 8.1. African Versus Asian Lineages: Molecular Adaptations and Epidemiological Relevance

Comparative studies indicate that African and Asian ZIKV lineages differ in replication kinetics, cytopathicity, and neurovirulence [41, 61]. In vitro investigations frequently demonstrate that African strains exhibit higher replication rates and greater cytotoxicity, whereas Asian‐lineage strains responsible for the epidemic in the Americas are more consistently associated with congenital malformations [10, 62]. Mutations in structural proteins such as prM and E have been proposed to enhance neurotropism and placental infection capacity [61].

However, many of these observations derive from cell culture systems or murine models employing supraphysiological viral inoculum, which may not accurately reflect the dynamics of human gestational infection [37, 64]. Epidemiological comparisons are further complicated by disparities in surveillance infrastructure, co‐circulating arboviruses, prior flavivirus immunity, and host genetic backgrounds [10, 78]. Consequently, although lineage‐specific molecular adaptations likely contributed to increased congenital pathogenicity, direct causal links between specific mutations and clinical outcomes remain incompletely resolved [41, 63].

### 8.2. Variability Among Experimental Models

A major limitation of the current literature lies in the diversity of experimental platforms used to study placental infection. In vitro trophoblast cultures allow detailed dissection of receptor usage, interferon signaling, and viral replication kinetics; however, they lack a three‐dimensional architecture and immune cell diversity [37]. Similarly, ex vivo placental explants preserve structural organization and cell–cell interactions; nevertheless, they are constrained by limited viability and substantial interindividual variability [61]. In addition, murine models enable genetic manipulation and controlled temporal analysis; however, species‐specific differences in placentation, interferon signaling pathways, and gestational immune response limit direct extrapolation to human pregnancy [64]. Finally, nonhuman primate models provide closer physiological similarity to humans, but their use is restricted by cost, ethical concerns, and small sample sizes [41].

### 8.3. Reproducibility and Conflicting Evidence

Discrepancies across studies may also reflect variation in gestational timing, viral strain selection, inoculum dose, and host immune status [41, 63, 64]. Interferon‐related findings are particularly sensitive to experimental design as outcomes differ markedly depending on whether Type I or Type III interferon pathways are selectively activated, inhibited, or genetically ablated [60, 64]. Similarly, reports of viral persistence vary according to sampling time points and detection methodologies, including reverse transcription quantitative polymerase chain reaction (RT‐qPCR) versus recovery of replication‐competent virus [41, 61].

Therefore, mechanistic claims regarding placental infection and vertical transmission must be interpreted within the specific experimental context in which they were generated. Integrative approaches combining standardized viral strains, human placental organoids, longitudinal clinical cohorts, and harmonized methodological frameworks are essential to improve reproducibility and reconcile conflicting findings. A more unified experimental strategy will be critical for translating mechanistic insights into predictive biomarkers and targeted therapeutic interventions [10, 58, 63, 64].

## 9. Conclusion

Integrated analysis of available evidence demonstrates that the placenta plays a dual and dynamic role as both a barrier and a target of ZIKV infection. The virus exploits structural and immunological vulnerabilities to establish sustained replication, selectively modulating antiviral and inflammatory responses to favor persistence and dissemination. The delicate equilibrium between coordinated interferon signaling and inflammatory amplification determines whether infection is contained or progresses toward placental dysfunction and vertical transmission. Thus, immune response intensity and timing emerge as central determinants of gestational outcome severity.

Despite significant advances, critical mechanisms driving vertical transmission remain incompletely elucidated. The precise determinants of placental barrier breakdown, the differential roles of Type I and Type III interferon across gestational stages, and the molecular signatures that predict fetal susceptibility require further clarification. Additionally, the extent to which the fetal compartment actively modulates placental antiviral responses remains poorly understood.

From a translational perspective, therapeutic modulation of interferon pathways represents both a promising and a challenging avenue. Strategies involving selective IFN‐λ enhancement or controlled attenuation of IFN‐I–driven inflammation must carefully balance antiviral protection with the risk of immune‐mediated placental injury. Development of maternal vaccination strategies and antivirals capable of targeting placental reservoirs without disrupting gestational immune tolerance also warrants further investigation.

In endemic regions, preexisting flavivirus immunity adds an additional layer of complexity. Cross‐reactive antibodies generated by prior exposure to DENV or other flaviviruses may influence ZIKV pathogenesis through antibody‐dependent enhancement or altered interferon dynamics, potentially modifying the susceptibility to vertical transmission. Understanding how cross‐immunity shapes placental immune responses is essential for vaccine design and public health strategies in flavivirus co‐endemic settings.

Future research integrating advanced placental organoid systems, longitudinal clinical cohorts, and multiomics profiling will be essential to resolve these uncertainties. Clarifying the immunological paradox at the maternal–fetal interface will not only deepen mechanistic understanding but also guide development of targeted preventive, diagnostic, and therapeutic interventions aimed at reducing the burden of CZS.

**Nomenclature**
ABCB1: ATP‐binding cassette subfamily B member 1
ABCG2: ATP‐binding cassette subfamily G member 2
BCL‐2: B‐cell lymphoma 2
CD4^+^: Cluster of differentiation 4‐positive
CD8^+^: Cluster of differentiation 8‐positive
CD14^+^: Cluster of differentiation 14‐positive
CNS: Central nervous system
CZS: Congenital Zika syndrome
DENV: Dengue virus
dNK: Decidual natural killer
E protein: Envelope protein
GBS: Guillain–Barré syndrome
HERVs: Human endogenous retroviruses
hPMCs: Human placental mesenchymal stromal cells
HMC‐1: Human mast cell line‐1
IFITM3: Interferon‐induced transmembrane protein 3
IFN‐I: Type I interferon
IFN‐III: Type III interferon
IFN‐α/β: Interferon alpha/beta
IFN‐λ: Interferon lambda
IgG: Immunoglobulin G
IL‐1β: Interleukin 1 beta
IL‐6: Interleukin 6
IL‐8: Interleukin 8
IL‐10: Interleukin 10
IRF3: Interferon regulatory factor 3
ISGs: Interferon‐stimulated genes
ISG20: Interferon‐stimulated gene 20
JAK/STAT: Janus kinase/signal transducer and activator of transcription
MAVS: Mitochondrial antiviral signaling protein
MDA5: Melanoma differentiation‐associated protein 5
miRNAs: Micro‐ribonucleic acids
mRNA: Messenger ribonucleic acid
NK: Natural killer
NS1–NS5: Nonstructural proteins 1–5
PRRs: Pattern recognition receptors
RIG‐I: Retinoic acid–inducible gene
IRNA: Ribonucleic acid
RT‐qPCR: Reverse transcription quantitative polymerase chain reaction
STAT2: Signal transducer and activator of transcription 2
TLR3: Toll‐like receptor 3
TLR7/8: Toll‐like receptors 7 and 8
TNF‐α: Tumor necrosis factor alpha
YFV: Yellow fever virus
ZIKV: Zika virus.

## Funding

This study was funded by the National Council for Scientific and Technological Development (CNPq), through the National Institute of Science and Technology (INCT), INCT‐VER project (Grant 406360/2022‐7) to Pedro Fernando da Costa Vasconcelos, and also received support from the Coordination for the Improvement of Higher Education Personnel (CAPES), Brazil.

## Ethics Statement

The authors have nothing to report.

## Consent

The authors have nothing to report.

## Conflicts of Interest

The authors declare no conflicts of interest.

## Data Availability

Data sharing is not applicable to this article as no datasets were generated or analyzed during the current study.
